# Sex-Based Differences Femoroacetabular Impingement and Hip Arthroscopy

**DOI:** 10.1007/s12178-025-09988-1

**Published:** 2025-08-06

**Authors:** Haley E. Smith, Andrea M. Spiker

**Affiliations:** Department of Orthopaedics and Rehabilitation, University of Wisconsin-Madison, 1685 Highland Ave, Madison, WI 53705 USA

**Keywords:** Hip arthroscopy, FAI, Labral tear, Sex differences

## Abstract

**Purpose of Review:**

Hip arthroscopy is an effective surgical procedure to treat intra-articular hip pathology including femoroacetabular impingement (FAI) and labral tears. This review aims to synthesize current evidence on sex-based differences in the pathology, presentation, surgical management, and outcomes of femoroacetabular impingement (FAI) and hip arthroscopy.

**Recent Findings:**

Emerging evidence indicates distinct morphological and clinical patterns of FAI between sexes. Cam-type morphology is more prevalent in males, whereas females more frequently present with pincer morphology and generalized joint hypermobility. Sex-specific differences in acetabular and femoral version, pelvic anatomy, and ligamentous laxity may contribute to variable symptomatology and diagnostic challenges. Postoperative outcomes following hip arthroscopy also appear to vary, with some studies reporting inferior outcome scores in females while others report no differences based on patient sex.

**Summary:**

Sex-based anatomical and biomechanical differences in FAI are clinically significant and may impact diagnosis, treatment strategy, and surgical outcomes. Recognizing and addressing these distinctions can optimize outcomes for both male and female patients. Continued research is needed to refine our understanding sex-specific etiology, pathology, and management approaches to ultimately improve long-term hip preservation.

## Introduction

Hip arthroscopy has become an increasingly common surgical intervention for a variety of intra-articular hip pathologies, offering a minimally invasive approach to address conditions such as femoroacetabular impingement (FAI), labral tears, and cartilage injury [1–3]. While the overall outcomes of hip arthroscopy are generally favorable, emerging evidence suggests that there may be important sex-based differences in the underlying pathology, clinical presentation, treatment response, and outcomes following this procedure [4–7].

Historically, sports medicine research and clinical practice have focused on male populations, leading to a potential underestimation of the unique experiences and needs of our female patients [8, 9]. However, a growing body of literature indicates that the etiology, hip biomechanics, and clinical manifestation of FAI and labral tears can differ significantly between sexes. For example, females may present with distinct patterns of FAI morphology, experience different types of labral tears, and exhibit varying degrees of ligamentous laxity compared to their male counterparts [4, 6, 7, 9]. These underlying biological and anatomical variations may influence patient presentation, surgical indications, technical considerations during arthroscopy, and the subsequent rehabilitation process.

Understanding these sex-specific factors are critical for optimizing patient care in hip arthroscopy. Recognizing differences in preoperative symptoms, radiographic and advanced imaging findings, surgical outcomes, and complication rates can enable clinicians to provide more tailored patient counseling and effective treatment strategies. This review aims to synthesize the current literature on sex differences in hip arthroscopy and FAI, exploring variations in epidemiology, etiology, clinical presentation, and postoperative outcomes. By highlighting these distinctions, we hope to foster a more nuanced understanding of how sex influences hip arthroscopy and ultimately contribute to improved outcomes for all individuals undergoing this procedure.

## Femoroacetabular Impingement Morphology

### Cam Morphology

Cam morphology result from an aspherical femoral head-neck junction, leading to abnormal contact with the acetabular rim during hip flexion and rotation. The development of cam morphology is closely tied to skeletal maturation. The adolescent period is particularly critical, as the proximal femoral physis is still open and susceptible to mechanical stress [6, 9, 10]. High-impact or high-volume activities, such as soccer, hockey, and basketball, have been implicated in stimulating abnormal physeal stress response and resultant cam formation. This stress likely disrupts the normal remodeling process at the head-neck junction, particularly in males who experience a later closure of the proximal femoral physis [9, 11].

**Fig. 1:**
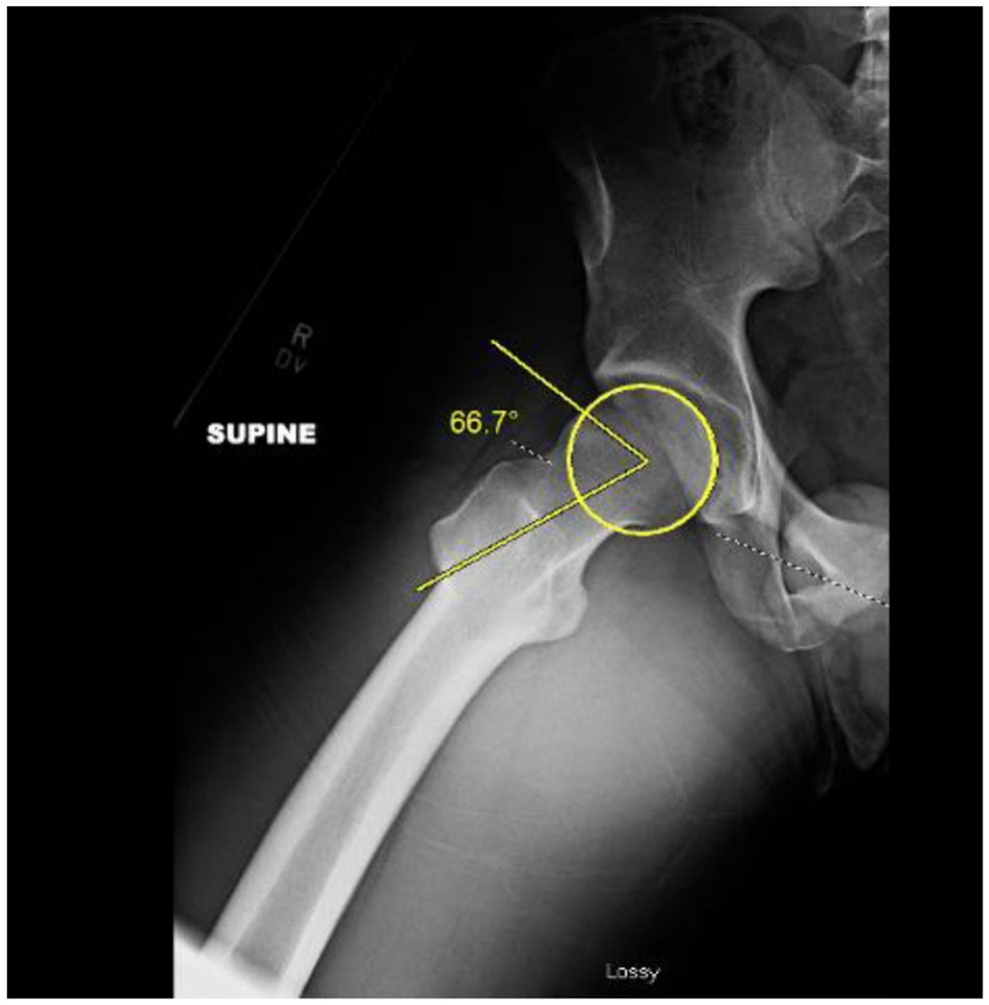
Alpha angle measured on 45^o^ Dunn Lateral radiograph

The alpha (α) angle describes where the head–neck junction loses sphericity. The alpha angle was classically measured on axial oblique magnetic resonance imaging (MRI) parallel to the plane of the femoral neck, can also be measured on plain radiographs and computed tomography (CT) Scans. The alpha angle is commonly characterized as pathologic when greater than 55°-60° [6, 12, 13]. (Fig. 1)

Epidemiological studies report a prevalence of cam morphology in approximately 20–30% of males in the general population, with rates 37% to over 90% observed in athletic cohorts in high risk sports [14–16]. In contrast, the prevalence in females is notably lower, typically ranging from 0 to 10% [9, 13] while in symptomatic females, the prevalence of cam morphology may be significantly higher [17]. There is large variability in the literature on sex disparities in cam morphology. Hooper et al. reported that male patients are nearly 40 times more likely to present with cam-type morphology than their female counterparts [18]; while other studies have suggested more balanced prevalence between sexes [13, 19].

When comparing male and female cam morphology, there is variation in size and location. In the study by Nepple et al., mean α angle was greater in men (70.8° vs. 57.6°) compared to women in patients requiring hip arthroscopy [17]. Yanke et al. performed a 3D analysis of cam deformities and compared male and female patients. They found that male cam height and volume were significantly larger than female cam lesions even when normalizing for femoral size. The positioning of the cam varied slightly. Female cam morphology may be less detectable on X-ray or 2D analysis [12]. Pathologic α-angle thresholds in females may need to be lowered compared to male patients to adequately capture female patients suffering from FAI [9, 13].

### Acetabular Morphology

In contrast to cam morphology, pincer-type impingement and coxa profunda are more commonly observed in females [15, 17, 20]. Pincer morphology, characterized by acetabular overcoverage of the femoral head as indicated by a high center-edge angle (greater than 40°, such as in coxa profunda or global overcoverage) or can also result from focal overcoverage [9]. Females have been shown to exhibit a higher prevalence of coxa profunda, which may contribute to increased contact between the femoral neck and acetabular rim during hip motion [21]. The sex-based difference in acetabular depth may predispose females to a different biomechanical pattern of impingement, potentially contributing to the higher prevalence of labral pathology seen in women with FAI.

**Fig. 2:**
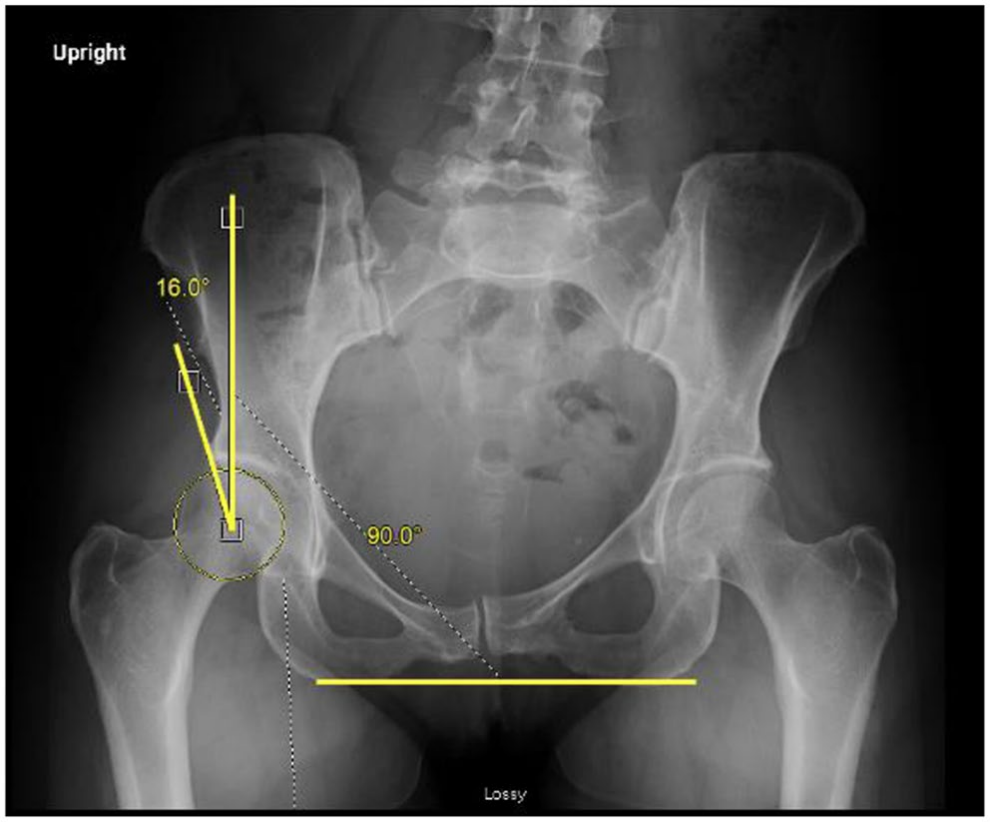
Measurement of the lateral center edge angle (LCEA) on a standing AP pelvis readiograph

Additionally, females are two to four times more likely to have developmental dysplasia of the hip and borderline dysplasia—conditions marked by insufficient acetabular coverage that may complicate the diagnosis and management of hip pain [9, 22]. Accurate identification of underlying dysplasia through careful review of clinical exam findings and imaging is critical for determining appropriate surgical indications and appropriately counseling patients on their postoperative expectations. A comprehensive discussion of dysplasia and its clinical implications, however, is beyond the scope of this review. (Fig. 2)

### Acetabular Version

**Fig. 3:**
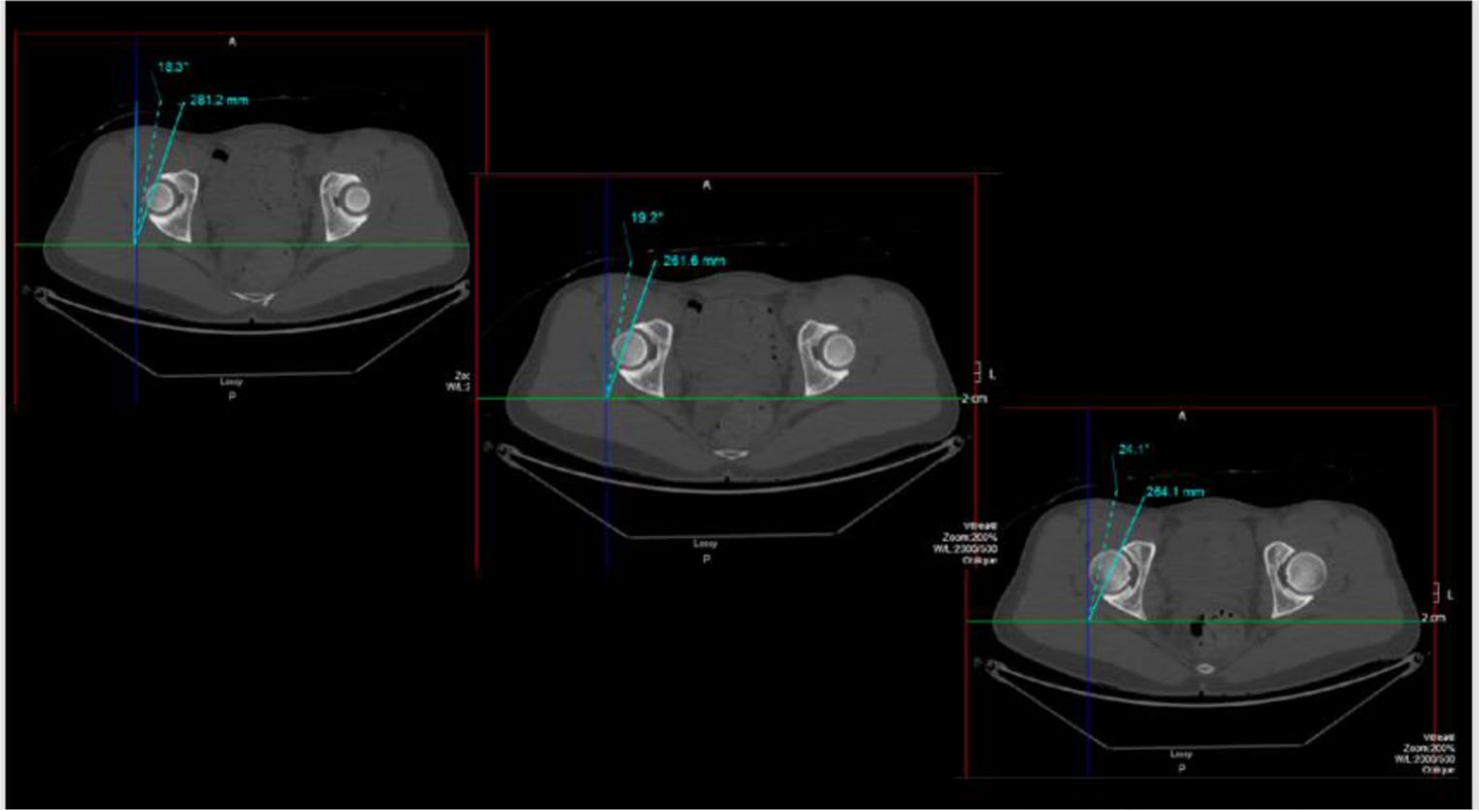
Acetabular version at the 1, 2 and 3 o’clock position measured off the axial CT slices of the acetabulum

Sex-based variation in acetabular version has also been documented, with females tending to have increased acetabular anteversion compared to males [23]. This increased anteversion results in greater anterior exposure of the femoral head and may influence impingement mechanics and labral stress patterns as well as the postoperative risk for instability. Hetsroni et al. suggest that acetabuloplasty between 12 and 3 o’clock needs to be more cautiously considered in women, to avoid increasing stresses at this weight-bearing zone [24]. Males, by contrast, more often present with relative or true acetabular retroversion, which can contribute to focal anterior overcoverage and dynamic impingement during flexion and internal rotation [25]. These differences in acetabular orientation may partially explain the sex-specific prevalence of cam versus pincer morphology [23]. (Fig. 3)

### Femoral Version

**Fig. 4:**
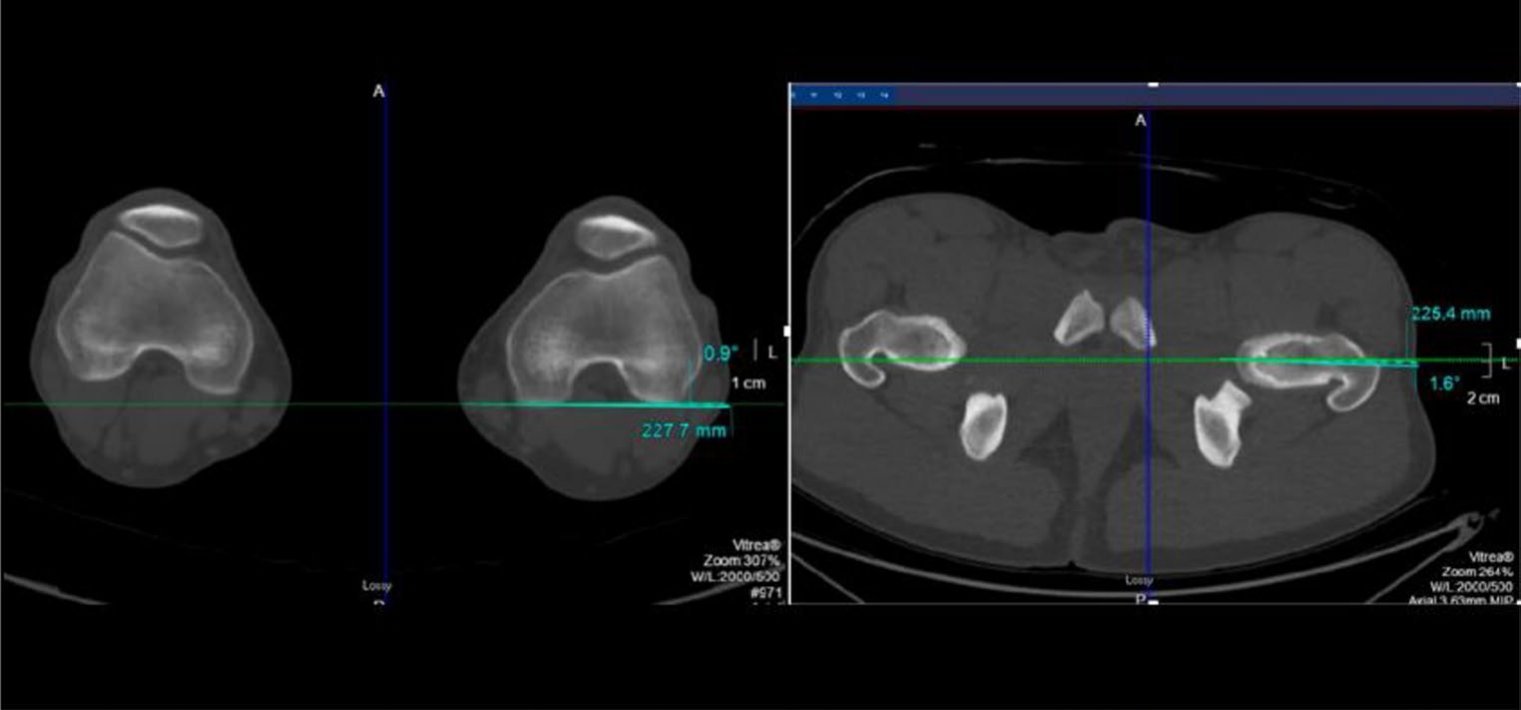
Measurement of the femoral version from the oblique axial femoral neck and axial distal femur slices of the CT

Differences in femoral version further complicate the biomechanical landscape of FAI. There is a growing body of literature on the interplay between femoral and acetabular version in FAI [26, 27]. On average, females demonstrate increased femoral anteversion compared to males [24]. Males typically have lower femoral version, which may exacerbate the effects of cam morphology and limit internal rotation, thereby increasing the likelihood of symptomatic impingement [24]. Fabricant et al. demonstrated that patients with relative femoral retroversion (< 5° femoral anteversion) may experience less improvement than those with normal or increased version [26]. (Fig. 4)

Understanding the hip morphology difference between the male and female patients is important and can potentially alter surgical planning and decision making.

### Intraoperative Findings and Procedures Performed

Numerous studies have demonstrated that cam deformities in males extended over a greater extent of the clockface and were associated with larger labral tears and chondral damage when compared to female patients [5, 7, 18, 28]. In a review of over 1400 patients, the Multicenter Arthroscopic Study of the Hip Group found several significant differences in the rates of procedures performed within each sex group. Specifically, male patients underwent femoroplasty, acetabuloplasty, acetabular chondroplasty, acetabular microfracture, and loose body removal at a higher rate than female patients, whereas female patients underwent trochanteric bursectomy, gluteus medius or minimus repair, and labral debridement at a higher rate than male patients [7].

### Clinical Presentation

The clinical presentation of hip pain associated with FAI can exhibit notable differences between sexes, potentially influencing diagnostic pathways and the perceived severity of the pathology.

While anterior groin pain exacerbated by hip flexion, adduction, and internal rotation (the FADIR test) is a common finding in both men and women with FAI, the nuances of symptom presentation, location, and associated complaints may vary [29]. Females are more likely present with an insidious onset of pain and with non-specific symptoms such as lateral hip, buttock, or back pain [4, 17, 20]. This can lead to diagnostic challenges and potentially delays in appropriate care for underlying FAI, as these pain patterns can overlap with other conditions such as greater trochanteric pain syndrome, lumbar spine pathology, sacroiliac joint dysfunction, and non-musculoskeletal etiologies [30]. Males more frequently report an acute, traumatic onset of pain and are more likely to have pain localized only to the anterior groin or “C-Sign” region [4].

Despite typically having less severe radiographic findings of FAI, females present with more severe pain and disability. A study from Clohisy et al. comparing 50 male and 50 female patients undergoing hip arthroscopy for symptomatic FAI found that females had significantly lower preoperative Modified Harris Hip Scores (mHHS), Hip Disability and Osteoarthritis Outcomes Score, and UCLA activity scores [15]. A large systematic review of over 200,000 patients across 74 studies also demonstrated that females present with more severe pain and disability on preoperative Visual Analog Scale pain scores and patient reported outcomes (PROs) [5].

On physical exam, male patients generally have more restricted hip range of motion at presentation. One study found that females had significantly more internal rotation ROM in flexion than males (16.4° vs. 6.9°, *p* < 0.001). Linder et al. found that females had significantly increased range of motion in flexion, abduction, internal and external rotation compared to the male patients [4]. Females are more likely to present with generalized ligamentous laxity (GLL), often assessed using the Beighton score. In a cohort of 2,701 patients undergoing primary hip arthroscopy for FAI and labral tears, 38.6% of females exhibited GLL (Beighton score ≥ 4), compared to 13.6% of males (*p* < 0.001) [31].

Multifactorial etiologies of pain that should be considered in the patient presenting with the chief complaint of hip pain. These can include gastrointestinal, genitourinary, pelvic floor, lumbar spine, core muscle, extra-articular hip conditions, in addition to intra-articular sources of pain. Given the high prevalence of asymptomatic labral tears and radiographic findings of FAI [32, 33], it is imperative that clinicians consider these other sources of pain in their differential. Sex-based differences should be considered in these other causes of hip pain as well. Due to pelvic bony morphology differences and the resulting force vectors of the rectus and adductor tendons, male patients are at increased risk for core muscle injury. Additionally, the embryological development of male sex organs results in a much higher incidence of inguinal hernias in the male patient population. The female pelvis which is slightly wider, but with smaller femoral offset, places females at increased risk for ischiofemoral impingement [34]. Females also experience greater trochanteric pain syndrome at a 4:1 ratio compared to males [30]. Recent literature also links pelvic floor pain and dysfunction to hip pain and pathology [35, 36]. This more commonly occurs in the female patient. If there is any question of the etiology of a patient’s pain, a thorough history should include the discussion of pelvic-floor symptoms including urinary and sexual symptoms [20].

### Hip Arthroscopy Complications

Following hip arthroscopy, common complications include but are not limited to superficial and deep infection, traction related nerve related injuries, heterotopic ossification, thromboembolic events, incomplete or over-resection of bony impingement, femoral neck fracture, persistent pain or mechanical symptoms, and gross or microinstability [37]. A systematic review and meta-analysis encompassing 74 studies with over 200,000 patients found that the relative risk of complications for females was 2.34 (95% CI, 1.33–4.10), indicating a significantly increased risk. Due to the heterogeneity of complications reported across studies in the systematic review, this particular study was unable to evaluate relative risk for individual complications [5]. Bedi et al. demonstrated that male patients were at increased risk for the development of heterotopic ossification. This may be related to the larger capsular cut and femoroplasty that is needed in many male patients undergoing hip arthroscopy [38]. Females are at increased risk for gross and microinstability following hip arthroscopy which may be due an increased prevalence of generalized ligamentous laxity and acetabular and femoral morphologic differences [37].

The need for revision arthroscopic surgery or conversion to total hip arthroplasty are often used as outcomes to define hip arthroscopy failure. Two large database studies identified female sex as a risk factor of revision surgery and conversion to total hip arthroplasty [39, 40]. Similarly, a review demonstrated that females were at increased risk for revision hip arthroscopy [41]. More recent studies have not consistently identified female sex as a predictor for the need for revision hip arthroscopy or conversion to arthroplasty [5, 42–44]. One of the reasons for the reported worse outcomes in female patients could be because these surgeries occurred before many of the sex-baesd differences in hip pathology had even been described. Surgeons may not have been aware of, and thus could not appropriately diagnose or treat, these sex-specific differences in female patients, leading to worse outcomes [45].

### Hip Arthroscopy Outcomes and Return-to-Sport

Early studies in modern hip arthroscopy consistently demonstrated significantly inferior postoperative PROs in female patients undergoing hip arthroscopy, prompting concerns regarding the clinical efficacy of this surgical intervention in women [46]. However, more recent literature has demonstrated that despite their lower preoperative scores, females demonstrate significantly greater improvements in PROs [5, 28, 47]. A large systematic review of 38 studies with 40,194 hip demonstrated no significant differences between sexes for postoperative PROs but males were more likely to achieve minimal clinically important difference (MCID) [48]. On the contrary, a machine learning model identified female sex as being a predictive factor for achieving MCID on PROs [49]. The lack of consensus across the literature on the sex-based differences in outcomes after hip arthroscopy was highlighted by the conclusion of McCormack et al. in their systematic review which demonstrated that one-third of the included studies determined that female sex was a negative predictor of postoperative outcomes, 13% found female sex to be a positive predictor, and 58% found no sex-based differences [42]. More equivalent PROs may be due to an improved understanding of FAI pathology, evolving surgical techniques, or larger and more numerous studies on outcomes following hip arthroscopy. It is difficult to ascertain the clinical relevance of sex-differences in PROs; Parker et al. comment on the importance of considering sex-specific trends in PROs in the evaluation of data [47, 50].

Similarly to PROs data, there is a lack of consensus across the literature on return to sport (RTS) after hip arthroscopy by sex. Dooley et al. examined a group of collegiate and professional athletes undergoing hip arthroscopy for FAI. They had an exceptionally high RTS rate at 91.9%. Of the athletes unable to return to sport, 73% were female [51]. However, Other studies have found higher rates of RTS in the female athletes suggesting that more severe intra-articular pathology at the time of hip arthroscopy may limit male athletes [52]. While rates may differ across studies, both male and female athletes demonstrate high rates of RTS, even at the collegiate and professional level [51, 52].

## Conclusions

In conclusion, the evidence reviewed highlights significant sex-based differences in FAI and hip arthroscopy. Male and female patients present with distinct patterns of FAI morphology and intra-articular pathology. Furthermore, the clinical presentation differs between male and female patients. Some studies suggest that outcomes following hip arthroscopy for FAI may not be uniform across sexes with variations in pain relief, functional improvement, time to reach clinically significant outcomes, and return to sport, however more research in this area is warranted as we learn more about the hip morphologic differences between the sexes.

Despite the growing body of literature, substantial gaps in our understanding of sex-based differences in FAI and hip arthroscopy persist. Future research is critical to elucidate the specific anatomic, biological, biomechanical, and clinical factors that drive these differences. Investigations into the influence of hormonal status, muscle activation patterns and sport specific biomechanics, and patient-reported outcome measures specific to each sex are warranted. Moreover, research focusing on optimizing surgical techniques and postoperative rehabilitation protocols tailored to the unique needs of male and female patients could lead to improved and more equitable outcomes. Ultimately, a deeper understanding of these sex-specific considerations will empower clinicians to provide more personalized and effective management strategies for both male and female patients undergoing hip arthroscopy for FAI.

## Key References

- Owen MM, Gohal C, Angileri HS, Hartwell MJ, Plantz MA, Tjong VK, et al. Sex-based differences in prevalence, outcomes, and complications of hip arthroscopy for femoroacetabular impingement: A systematic review and Meta-analysis. Orthop J Sports Med. 2023;11:23259671231188332.
-
  - A recent systematic review and meta-anaylsis of over 200,000 hip arthroscopy patients which highlighted the differences in FAI pathology between male and female patients and the PRO improvements in female patients.
- Salvo JP, Nho SJ, Wolff AB, Christoforetti JJ, Van Thiel GS, Ellis TJ, et al. Sex-dependent differences in preoperative, radiographic, and intraoperative characteristics of patients undergoing hip arthroscopy: Results from the multicenter arthroscopic study of the hip group. Arthroscopy. 2018;34:844–52.
-
  - A retrospective multicenter study highlights the sex differences in femoroacetabular impingement in pre-operative presentation, radiographic findings, and surgical procedures performed.

## Data Availability

No datasets were generated or analysed during the current study.
